# Nanoarchitectonics of photothermal materials to enhance the sensitivity of lateral flow assays

**DOI:** 10.3762/bjnano.14.82

**Published:** 2023-10-04

**Authors:** Elangovan Sarathkumar, Rajasekharan S Anjana, Ramapurath S Jayasree

**Affiliations:** 1 Division of Biophotonics and Imaging, Biomedical Technology Wing, Sree Chitra Tirunal Institute for Medical Sciences and Technology, Poojappura, Thiruvananthapuram-695012, Kerala, India

**Keywords:** lateral flow assay, nanoarchitectonics, photothermal, photothermal nanomaterials, signal amplification

## Abstract

Lateral flow assays (LFAs) are currently the most widely used point-of-care testing technique with remarkable advantages such as simple operation, rapid analysis, portability, and low cost. Traditionally, gold nanoparticles are employed as tracer element in LFAs due to their strong localised surface plasmon resonance. However, this conventional LFA technique based on colorimetric analysis is neither useful to determine critical analytes with desired sensitivity, nor can it quantify the analytes. Various signal amplification strategies have been proposed to improve the sensitivity and the quantitative determination of analytes using LFAs. One of the promising strategies is to enhance the photothermal properties of nanomaterials to generate heat after light irradiation, followed by a temperature measurement to detect and quantify the analyte concentration. Recently, it has been observed that the nanoscale architecture of materials, including size, shape, and nanoscale composition, plays a significant role in enhancing the photothermal properties of nanomaterials. In this review, we discuss the nanoarchitectonics of nanomaterials regarding enhanced photothermal properties and their application in LFAs. Initially, we discuss various important photothermal materials and their classification along with their working principle. Then, we highlight important aspects of the nanoscale architecture (i.e., size, shape, and composition) to enable maximum light-to-heat conversion efficiency. Finally, we discuss some of the recent advances in photothermal LFAs and their application in detecting analytes.

## Introduction

Lateral flow assays (LFAs) are a versatile and convenient point-of-care testing (POCT) technique used to detect analytes of interest in a wide range of applications such as diagnostics, environmental monitoring, and food quality measurements [1–3]. LFAs are well known for their simplicity, affordability, accessibility to patients, and ease of use [4]. Since the discovery of this technique in the 1980s, it has been considered as a direct approach to detect the presence or absence of analytes with the concept of “sample to result” [5]. The principle of LFAs is the formation of an antigen–antibody–tracer complex at the test line in the presence of analytes. Generally, LFAs consists of four different parts, namely sample pad, conjugate pad, nitrocellulose membrane, and adsorbent pad, maintaining a smooth flow of reagents through the LFA membrane. In the presence of a sample, the analyte initially reacts with affinity molecules, commonly antibodies linked with tracers such as gold nanoparticles, before being captured by immobilized biomolecules on a test line in the nitrocellulose membrane [6–7].

In conventional LFAs, most commonly gold nanoparticles and coloured cellulose nanobeads have been used as tracer elements because of strong light absorption and surface plasmon resonance, which yield the coloured signal of the test line [8]. A colour change of the test line indicates the presence of an analyte in the sample [1]. In the point-of-care context, traditional LFAs have been developed for qualitative and/or semi-quantitative analyses of biological samples. Though LFAs have advantages over other POCT techniques, there is always a demand for improvement because LFAs are insufficient to detect analytes at low concentrations (i.e., below ng/mL) [9]. At such low concentrations, the change of colour of the test line cannot be distinguished by the naked eye. LFAs give a test result with “yes” or “no” answer, and more sophisticated tests are required to validate the results [6]. Additionally, LFAs suffer from limitations such as lack of quantitative determination of analytes and appropriate signal amplification techniques to see the test line at very low concentrations of analytes. Thus, the test results from LFAs are not comparable with standard centralized laboratory techniques such as enzyme-linked immunosorbent assay, real-time polymerase chain reaction, and high-pressure liquid chromatography, which have a detection limit from nanomolar to attomolar concentrations, whereas the detection limit of LFAs is only between millimolar and micromolar concentrations [10]. To overcome this, various modifications of LFAs have been explored [11–12]. For example, Petrakova et al. used different architectures of gold nanoparticles in LFAs to improve the sensitivity of the assays [11]. However, even with these modifications, it relied on the colorimetric principle and was not applicable for the quantitative determination of analytes.

In recent years, to address these challenges, various signal amplification strategies, such as DNA amplification, nanozyme activity, surface-enhanced Raman spectroscopy (SERS), fluorescence, ultrasound, and photothermal methods, have been tried in LFAs [4,13–14]. For example, fluorescence-based LFAs exhibited a 100- to 1000-fold higher sensitivity than conventional LFAs [15]. However, working with fluorescence-based LFAs is complex because of photoinstability, bleaching, and degradation. Gold nanoparticles possess intrinsic peroxidase-like activity, which converts particular peroxidase substrates into coloured products in the presence of hydrogen peroxide. The enzyme-like properties of nanomaterials have been utilized in various LFA formats. The inclusion of additional peroxidase substrates in LFAs increased the detection limit from the nanogram to the picogram level [16–17]. Various tracer elements have been developed to increase the sensitivity of an assay, including noble metal nanomaterials, metal oxides, plasmonic nanostructures, carbon-based materials, and magnetic materials [18–19]. Among them, noble metal nanostructures are a new class of photothermal nanomaterials with favourable characteristics to increase the sensitivity of an assay. For example, gold nanoparticles (GNPs) can generate a strong heat signal in addition to the visual colour change [20–21]. Generally, photothermal nanomaterials are being used in cancer therapy, removal of bacterial biofilms, and sensing applications [22–24]. Photothermal nanomaterials produce heat in response to the irradiation of photons at a particular wavelength [23].

**Figure 1:**
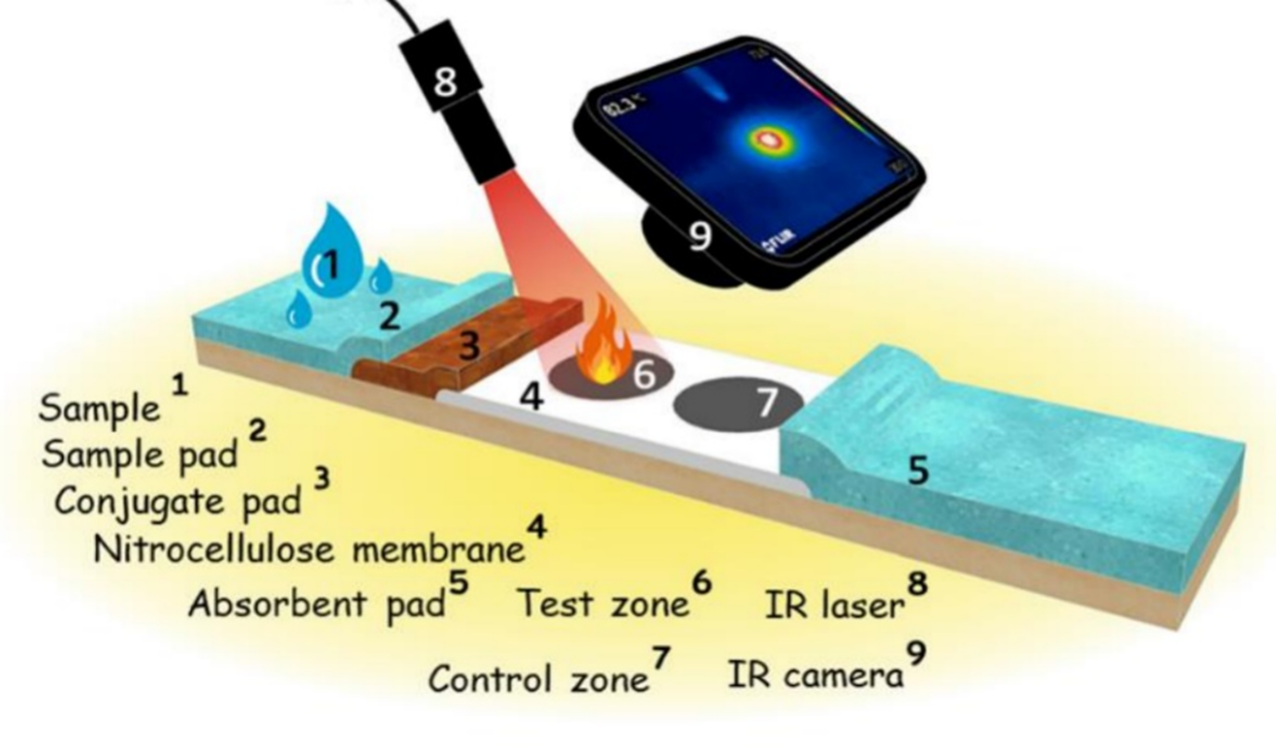
Schematic representation of photothermal LFA (Figure 1 was reprinted from [27], Colloids and Surfaces B: Biointerfaces, vol. 186, by V. Shirshahi; S. N. Tabatabaei; S. Hatamie; R. Saber, “Photothermal enhancement in sensitivity of lateral flow assays for detection of E-coli O157:H7”, article no. 110721, Copyright (2019), with permission from Elsevier. (This content is not subject to CC BY 4.0.)

Similarly, when plasmonic nanoparticles are exposed to light of a specific wavelength, they undergo coherent oscillation of surface electrons, leading to the production of thermal energy and enhanced electromagnetic signals. This phenomenon is known as localized surface plasmon resonance (LSPR), which has been recognized as a reliable technique to enhance the sensitivity of LFAs. In LFAs, the nanomaterials are deposited in the test zone along with the analyte to be determined. Upon laser irradiation, thermal energy is generated. The thermal output can be quantified using infrared thermal cameras or thermometers [25]. The quantity of heat produced is directly proportional to the amount of nanomaterial present in the test zone, which, in turn, is proportional to the concentration of the analyte in the sample (Figure 1). The integration of light energy in the procedure does not complicate the assay; its native procedure and rapidness are maintained. Further, the development of a laser readout system to measure the signal intensity of the test line could potentially bring out the characteristic feature of POCT [10]. To establish a correlation between the nanoscale architecture and the optical properties of materials in order to enhance the sensitivity is an important research area. The nanoscale architecture, including shape, size, composition, and surface properties, is expected to play a major role in the photothermal properties of nanomaterials [26]. The nanoscale architecture is not only responsible for the optical characteristics of the nanomaterials; it also influences the capillary movement of nanoparticles inside the nitrocellulose membrane pores.

**Figure 2:**
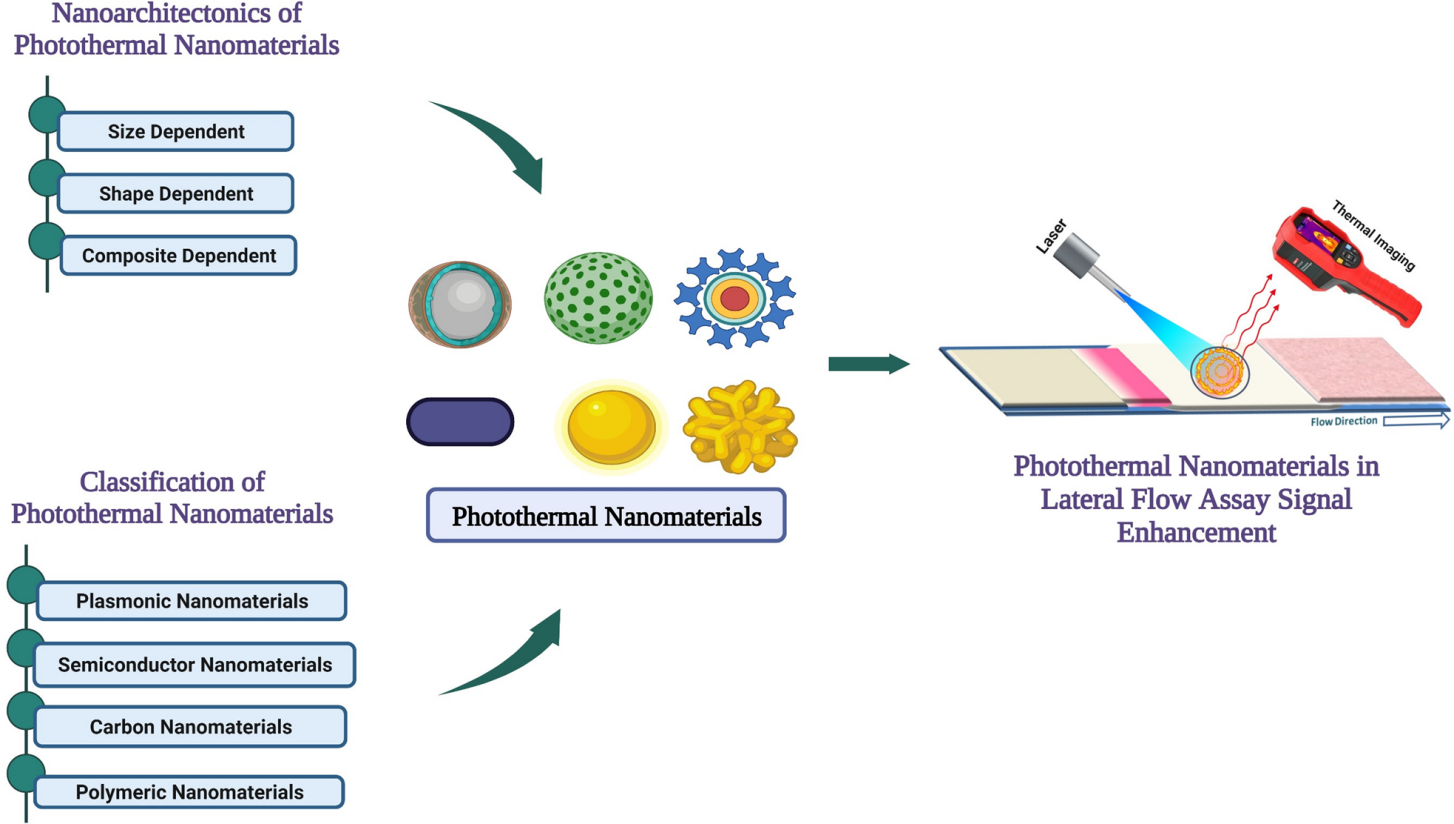
Concise overview of enhancing sensitivity in lateral flow assays with photothermal nanomaterials. (Figure 2 was created with https://biorender.com/. This content is not subject to CC BY 4.0.)

Herein, we discuss the current development of photothermal nanomaterials regarding the application in LFAs. As an introduction, we discuss the classification of different photothermal nanomaterials, including noble metal nanomaterials, semiconductor nanomaterials, carbon-based nanomaterials, and polymeric nanomaterials. Then, we review the unique nanoscale architecture of nanomaterials responsible for their photothermal properties. Finally, we explore the current status of photothermal LFA systems and their development in POCT techniques. We also try to highlight the importance of photothermal nanomaterials in signal amplification techniques, which might allow for the sensitive and quantitative detection of analytes in LFAs and other POCT devices. A graphical outline of the content of this review is provided in Figure 2.

## Photothermal nanomaterials

The photothermal properties of nanomaterials have been extensively studied for therapeutic applications. Heat generation under exposure to near-infrared (NIR) light is commonly used against deadly diseases such as cancer [28–30]. Because of the NIR absorption potential, nanomaterials can also be used in the field of photothermal nanosensors, which is an underexplored, yet highly potential, area of research [31]. To use nanoparticles for photothermal applications, the absorption wavelength should lie in the NIR region (650–900 nm). Different types of nanoparticles are used, depending on their physical and chemical properties [28].

### Classification of photothermal nanomaterials

**Figure 3:**
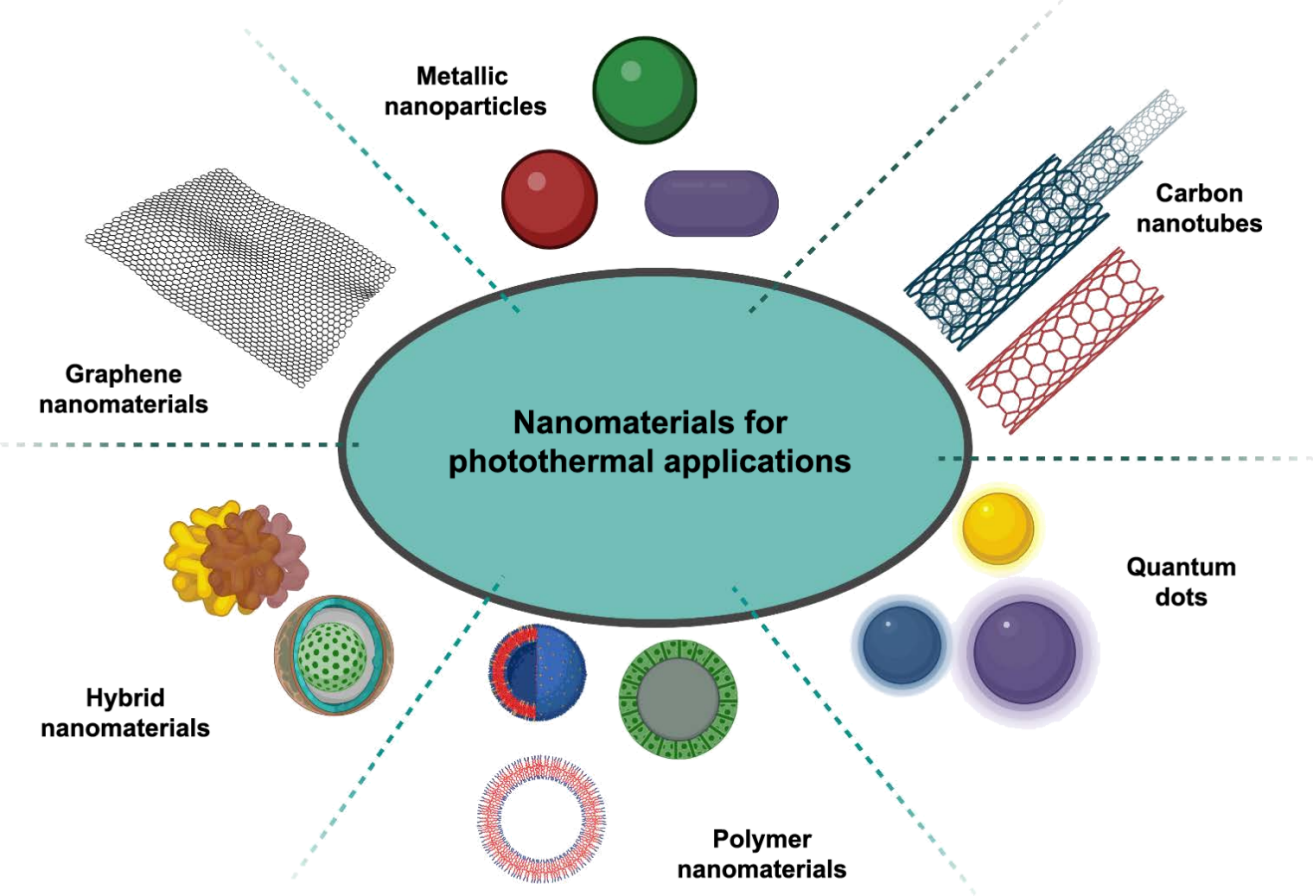
Schematic illustration of important photothermal materials (Figure 3 was created with https://biorender.com/. This content is not subject to CC BY 4.0.)

The photothermal properties of nanomaterials mainly depend on chemistry and architecture of the materials [32]. Nanomaterials exhibiting NIR absorption and possessing well-engineered characteristics in terms of size, shape, and composition are regarded as potential candidates for diverse photothermal applications, including photothermal LFAs. Photothermal materials can be categorized into four main types, depending on their chemistry and light absorption properties, that is, plasmonic materials (e.g., Au, Ag, and Pt), semiconductor materials (e.g., transition metal oxides, transition metal chalcogenides, and transition metal dichalcogenides), carbon-based nanomaterials (such as graphene oxide and carbon nanotubes), and polymer nanomaterials [33–34] (Figure 3). The subsequent sections offer a comprehensive discussion on the photothermal properties of these materials. These photothermal materials hold significant promise in enhancing the sensitivity and effectiveness of photothermal LFA and various other applications.

**Plasmonic photothermal nanoparticles:** The optical, physical, and chemical properties of plasmonic nanoparticles highly depend on the number of valence electrons. When the oscillating frequency of the electron cloud matches the frequency of the electromagnetic irradiation, LSPR occurs [35]. The excited plasmonic photothermal agent decays to the ground state after internal conversion by emitting the excess energy absorbed in two different ways, namely a radiative pathway, which will enhance the electric field, and a non-radiative pathway, during which radiation is emitted as heat [36].

**Figure 4:**
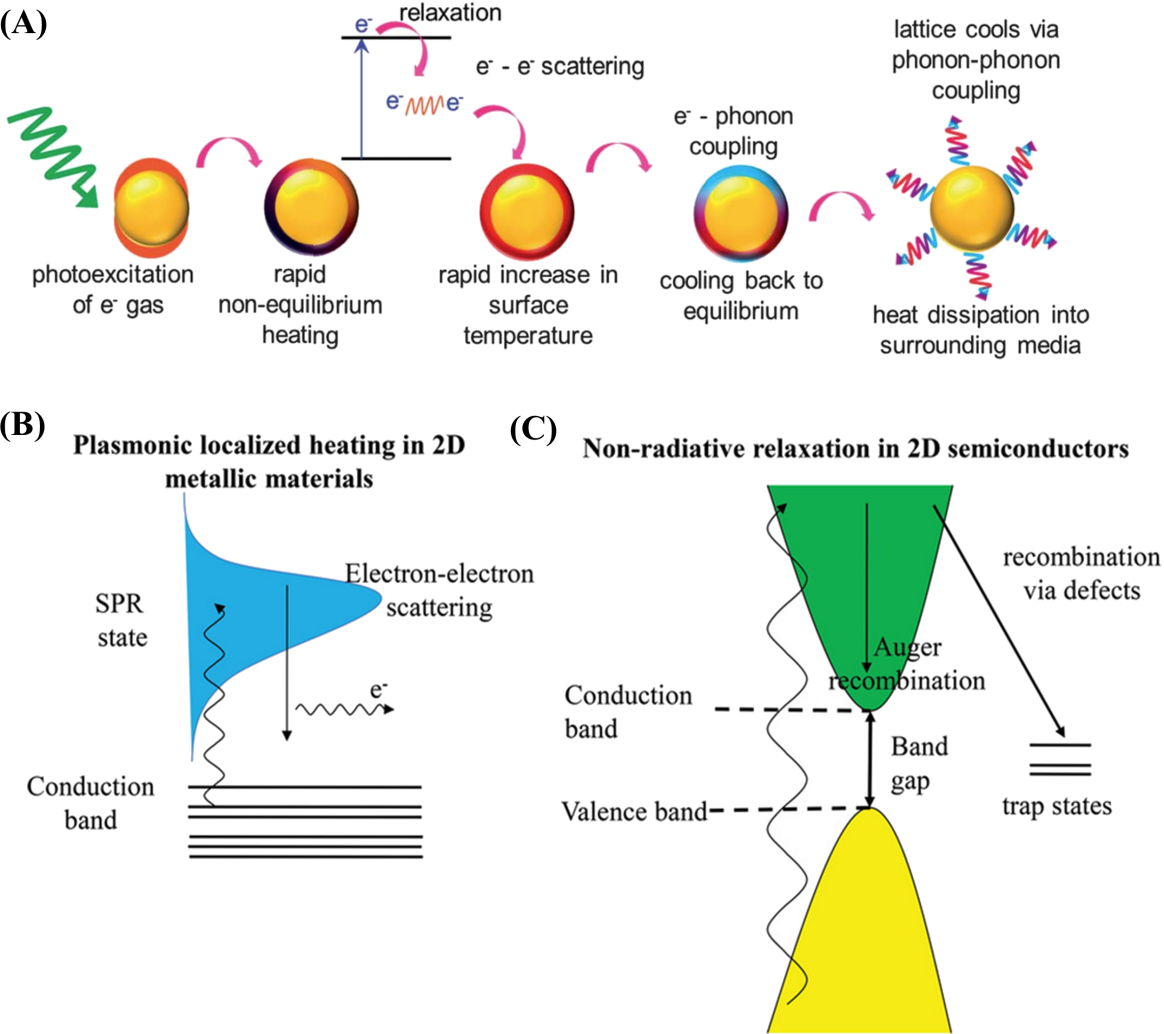
Heat conversion by plasmonic nanoparticles (A & B), (Figure 4A was republished with permission of the Royal Society of Chemistry, from [37], "Emerging advances in nanomedicine with engineered gold nanostructures" by J. A. Webb and R. Bardhan, Nanoscale, vol. 7, issue 6, © 2014, permission conveyed through Copyright Clearance Center, Inc. This content is not subject to CC BY 4.0.) Non-radiative mechanism in 2D semiconductor (C). (Figure 4B and C was reproduced from [38] (© 2020 Z. Xie et al., published by WILEY-VCH Verlag GmbH & Co. KGaA, distributed under the terms of the Creative Commons Attribution 4.0 International License, https://creativecommons.org/licenses/by/4.0)).

In the non-radiative decay, three processes are likely to occur, namely (i) the relaxation of electrons from non-Fermi levels to the Fermi level (electron–electron scattering), (ii) the cooling of hot electron gas through electron–phonon scattering, and (iii) the emission of heat to the surrounding (phonon–phonon scattering) (Figure 4A,B). The LSPR effect highly depends on size, shape, composition, interparticle distance, dielectric constant, and surrounding medium of the particles [36–37].

While Pt, Ag, and Au particles all exhibit photothermal properties, Au nanoparticles are commonly used for photothermal applications. By changing the architecture of the gold nanoparticles, their plasmonic absorption can be tuned from the visible to the NIR region. Among the different structures of gold, gold nanorods are commonly used as photothermal agents because of the tuneable aspect ratio, anisotropic architecture, and high photoconversion efficiency (PCE). However, studies report that gold nanorods suffer from poor photostability, and loss of shape and NIR LSPR upon laser irradiation [39]. By using palladium nanostructures, photothermal stability could be achieved to some extent as they did not change their shape upon laser irradiation. Palladium nanosheets exhibited a high PCE of 52% at 808 nm [40]. Palladium nanocorollae have shown a photothermal effect due to NIR LSPR with a temperature increase up to 50 °C [36].

**Semiconductor nanomaterials:** Generation and relaxation of electron–hole pairs are responsible for heat generation in semiconductor nanomaterials. When excited by photons with an energy greater than or equal to the bandgap of the materials, electron–hole pairs are generated. The electrons excited to the conduction band will leave holes in the valence band. The relaxation of these electrons can occur radiatively in the form of photons and non-radiatively in the form of phonons. The phonons generate heat in these materials [36] (Figure 4C).

Transition metal sulfide nanomaterials gain much attention in photothermal applications, because of their low cost, high biocompatibility, and considerably high PCE. Nanoparticles of copper sulfide and tungsten oxide generate heat with maximum conversion efficiency. Chen et al. reported that PEGylated W_18_O_49_ nanowires are able to enhance the absorption of light in the NIR range when irradiated with a 980 nm laser with an increase in temperature up to 40 °C within a short period of time [41].

Transition metal chalcogenides are another class of photothermal nanomaterials that exhibit strong NIR absorption, good photostability, and low toxicity. The architectonics of these nanomaterials also plays an important role regarding the PCE. Among the different types of transition metal oxide nanoparticles, iron oxide nanoparticles are the most prominent ones because of their biocompatibility, low toxicity, ease of synthesis, and high photothermal conversion efficiency. The influence of a magnetic field can also increase temperature generation by such nanoparticles, which is called magnetic hyperthermia. Since iron oxide nanoparticles absorb in the NIR region, irradiating them with a proper source will produce heat, which makes them suitable for theranostic purposes [32,42–43].

**Carbon nanomaterials:** Carbon-based materials have been identified as promising candidates for photothermal applications because of their remarkable ability to absorb light across a broad spectrum and their high PCE. The main classes of photothermal carbon-based nanomaterials are carbon nanotubes, graphene-based nanomaterials, carbon quantum dots, and carbon-based composite materials. Among these, carbon nanotubes have the highest photothermal conversion efficiency. In addition, these materials also possess high thermal and electrical conductivity, high aspect ratio, light weight, and high mechanical strength, because of which these materials are used for photothermal applications [36].

Polyhydroxylated fullerenes were found to be suitable candidates for photothermal applications when irradiated with high-power lasers (100 W/cm^2^) because of the cage-like structure. This particular carbon allotrope is very stable under NIR light [32]. Graphene-based nanoparticles exhibited better photothermal properties when used with a low-power laser source. In a multimodal system of gold, iron oxide, and graphene oxide (GO) nanoparticles, the photothermal properties of graphene, the magnetic properties of iron oxide, and the surface plasmon resonance of gold enable multiple functions in a single system. The presence of gold nanoparticles in the composite can increase the light absorption through surface plasmon resonance. Hence, graphene-based multifunctional nanocomposite materials could be employed as promising photothermal agents [44].

**Polymer-based nanomaterials:** Because of high biocompatibility, high PCE, and facile synthesis, polymers such as polyaniline (PANI), poly(3,4-ethylenedioxythiophene) (PEDOT) and polydopamine (PDA) are used as photothermal agents. After protonation in acidic environments, PANI absorbs photons in the NIR range. PEDOT maintains its photothermal properties even after continuous heating or cooling [24].

NIR light irradiation for 10 min can yield temperatures up to 61 °C in mesoporous PDA with a photoconversion efficiency of 26.7%. Despite the advantages, many of the polymer nanoparticles show strong photoluminescence and do not withstand long-term light irradiation. To overcome this challenge, hybrid materials with other functional materials have been found to be useful. Covalent organic frameworks (COFs) are a new class of organic polymers containing B, C, Si, N, and O in their backbones. Conjugation of COFs with Fe_3_O_4_ nanoparticles will yield a two to three times higher PCE than that of bare Fe_3_O_4_ nanoparticles. COF–CuSe can also increase the photothermal conversion efficiency up to 26%, which is higher than that of COF-Fe_3_O_4_ [36].

### Nanoarchitectonics of photothermal materials

The photon-to-heat conversion efficiency of nanomaterials depends on intrinsic factors, such as shape, size, crystallinity, and the aggregation pattern of nanomaterials, and extrinsic factors, including surrounding medium, medium viscosity, and concentration of the nanomaterial. Most often, nanomaterials with complex three-dimensional structures have better optical and physicochemical properties for the use in colorimetry, SERS, fluorescence, and photothermal applications [45]. For instance, compared to spherical architectures, anisotropic nanoscale architectures, such as nanostars, nanoflowers, nanopopcorns, nanorods, and nanocages, exhibit superior SERS and photothermal conversion activities [46–47]. Therefore, it is important to construct the nanomaterials with specialized nanoscale architectures to ensure maximum photothermal conversion efficiency. In this section, we will compare three main nanoscale architecture aspects, namely size, shape, and composition, which influence the photothermal properties and the application in photothermal LFA.

#### Size-dependent photothermal properties

**Figure 5:**
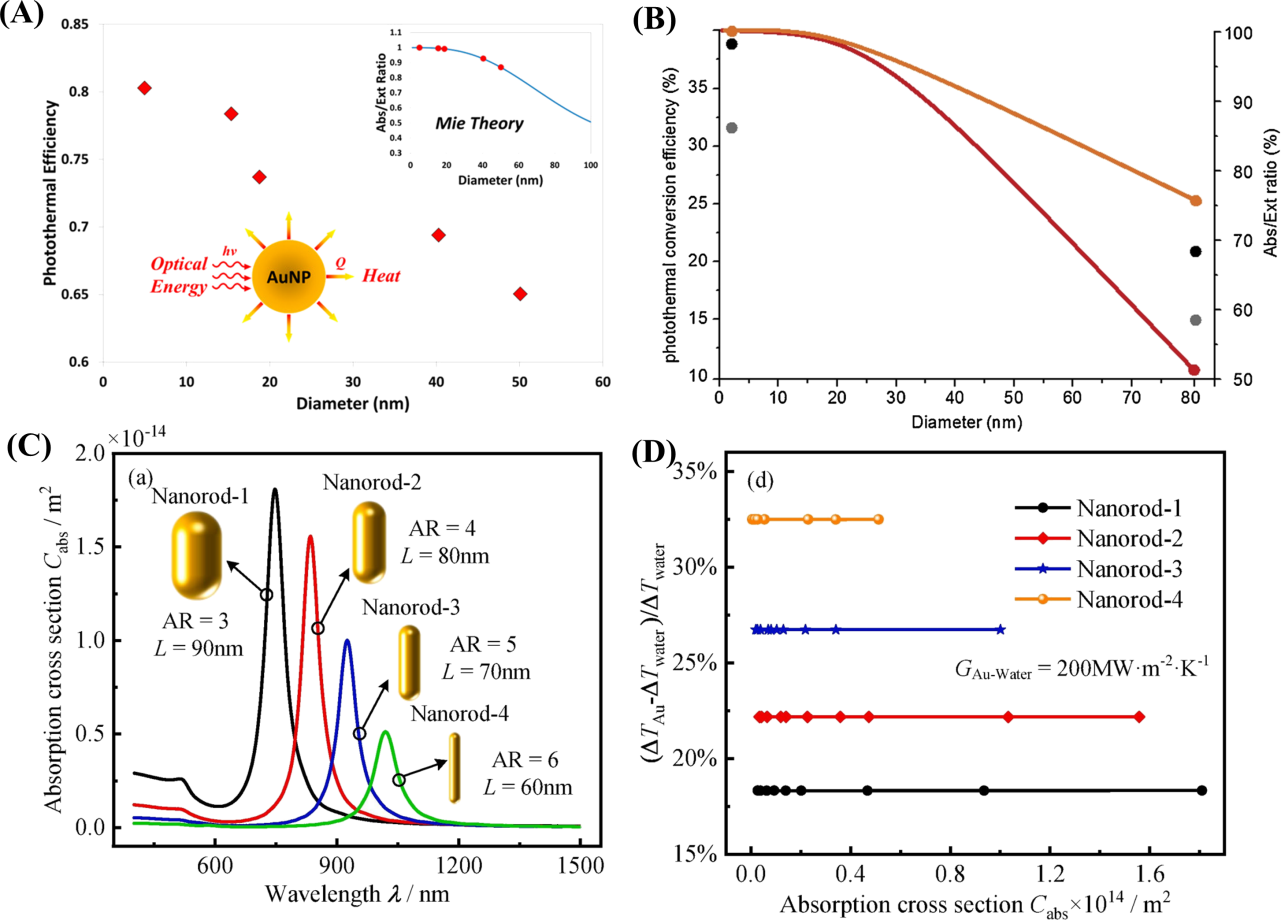
Photothermal conversion efficiency of Au nanoparticles with different diameters (5–50 nm) (A). (Figure 5A was reprinted with permission from [53], Copyright 2013 American Chemical Society. This content is not subject to CC BY 4.0.). Photothermal conversion efficiency of 2 and 8 nm platinum nanoparticles. Orange and the red line represent 2 and 80 nm Pt nanoparticles respectively and their calculated abs/ext ratio (B). (Figure 5B was reprinted from [54], Photodiagnosis and Photodynamic Therapy, vol. 29, by J. Depciuch; M. Stec; B. Klebowski; A. Maximenko; E. Drzymała; J. Baran; M. Parlinska-Wojtan, “Size effect of platinum nanoparticles in simulated anticancer photothermal therapy“, article no. 101594, Copyright (2019), with permission from Elsevier. This content is not subject to CC BY 4.0.). Comparison of absorption spectra of different sizes Au-nanorods (C). Maximum temperature raising profile with respect to absorption cross-section of Au-nanorods (D). (Figure 5C and D was reproduced from [48] (© 2022 J.-P. Sun et al., published by Elsevier, distributed under the terms of the Creative Commons Attribution-NonCommercial-NoDerivatives 4.0 International License, https://creativecommons.org/licenses/by-nc-nd/4.0/). This content is not subject to CC BY 4.0.)

Gold nanoparticles have benefits in the field of diagnostics because of their high absorption cross section, LSPR, ease of preparation, stability, and variable surface chemistry [48]. Most importantly, the high number of synthesis methods to control the nanoscale architecture makes them a great choice for biomedical applications [49–50]. Gold nanostructures such as spheres, rods, stars, shells, and cages are often used as photothermal agents [51–52]. The surface plasmon resonance frequency of gold nanomaterials can be easily controlled by size, shape, and aspect ratio of the nanomaterials. The size-dependent thermal conversion efficiency of gold nanospheres was studied using 5 to 50 nm particles under 532 nm laser light irradiation [53] (Figure 5A). The thermal conversion efficiency increased from 0.65 to 0.803 on decreasing the particle size from 50 to 4.98 nm. In another study, the size-dependent photothermal conversion efficiency of platinum nanomaterials was studied by Depciuch et al. for cancer therapy. Spherical platinum nanoparticles with diameters of 2 and 80 nm were studied regarding the photothermal activity in colon cancer cells under 650 and 808 nm laser irradiation [54] (Figure 5B). The nanoparticles with a size of 2 nm showed a 5–6% higher photothermal conversion efficiency than the 80 nm particles. The higher photothermal effect of smaller nanoparticles can be explained by the Mie theory, which states that as nanomaterials become larger, they deflect more light than they absorb, resulting in reduced photothermal conversion [53]. This is because the ratio between absorption coefficient (µ_a_) and scattering coefficient (µ_s_) is higher for smaller particles. Compared to the 80 nm gold nanoparticles (µ_a_ = 67.88 µm^−1^), 40 nm GNPs have a fivefold higher cross-sectional area, hence, they absorb more light (µ_a_ = 87.36 µm^−1^) than they scatter (µ_s_ = 5.25 µm^−1^) and yield a great amount of heat [55]. The cross-sectional area and, hence, the absorption efficiency also depend on the architecture of the nanomaterial. Absorption-to-scattering ratios of gold nanorods and gold nanoshells are high compared to that of gold nanoparticles and can be further increased by changing the dimensions or the cross-sectional areas [56–57]. Light scattering of nanomaterials should be minimum for an efficient photon-to-heat conversion. The photothermal heat conversion efficiency of gold nanorods with smaller diameter (radius = 11.43 nm, µ_a_ = 1003.87 µm^−1^) is higher than that of larger gold nanorods (radius = 21.86 nm, µ_a_ = 449.34 µm^−1^) [55]. The photon-to-heat conversion efficiency of gold nanorods is twice that of gold nanoshells because of the higher cross-sectional area. The PCE of the gold nanoshells can be increased by either increasing the core-to-shell radius ratio or by increasing their aspect ratio [58–59]. In a theoretical study, the size-dependent optical properties of three different nanomaterials, including gold nanospheres, gold nanorods, and gold nanoshells, were calculated. Gold nanorods showed high absorption-to-scattering ratios [55]. Especially smaller nanorods showed excellent absorption-to-scattering ratios in the NIR region, making them a favourable candidates for optical and photothermal applications. A smaller diameter of nanorods makes them excellent light absorbers for photothermal applications, while greater dimensions makes them efficient light scatterers for imaging applications [57]. The absorption and photothermal characteristics of four differently sized nanorods were analysed by Sun and co-workers. The results again verified that smaller nanorods have better heat transfer characteristics than larger nanorods because of their high cross-sectional area [48] (Figure 5C,D).

Though the application of photothermal nanomaterials is not explored much in LFAs, it is expected that enhanced plasmon resonance could be a major strategy to enhance the efficiency. This could be achieved by varying aspect ratio, material composition, and shape to yield better analytical performance in LFAs [60]. In addition, the size of the nanomaterial is also important for capillary movement and smooth flow in the nitrocellulose membrane.

#### Shape-dependent photothermal properties

**Figure 6:**
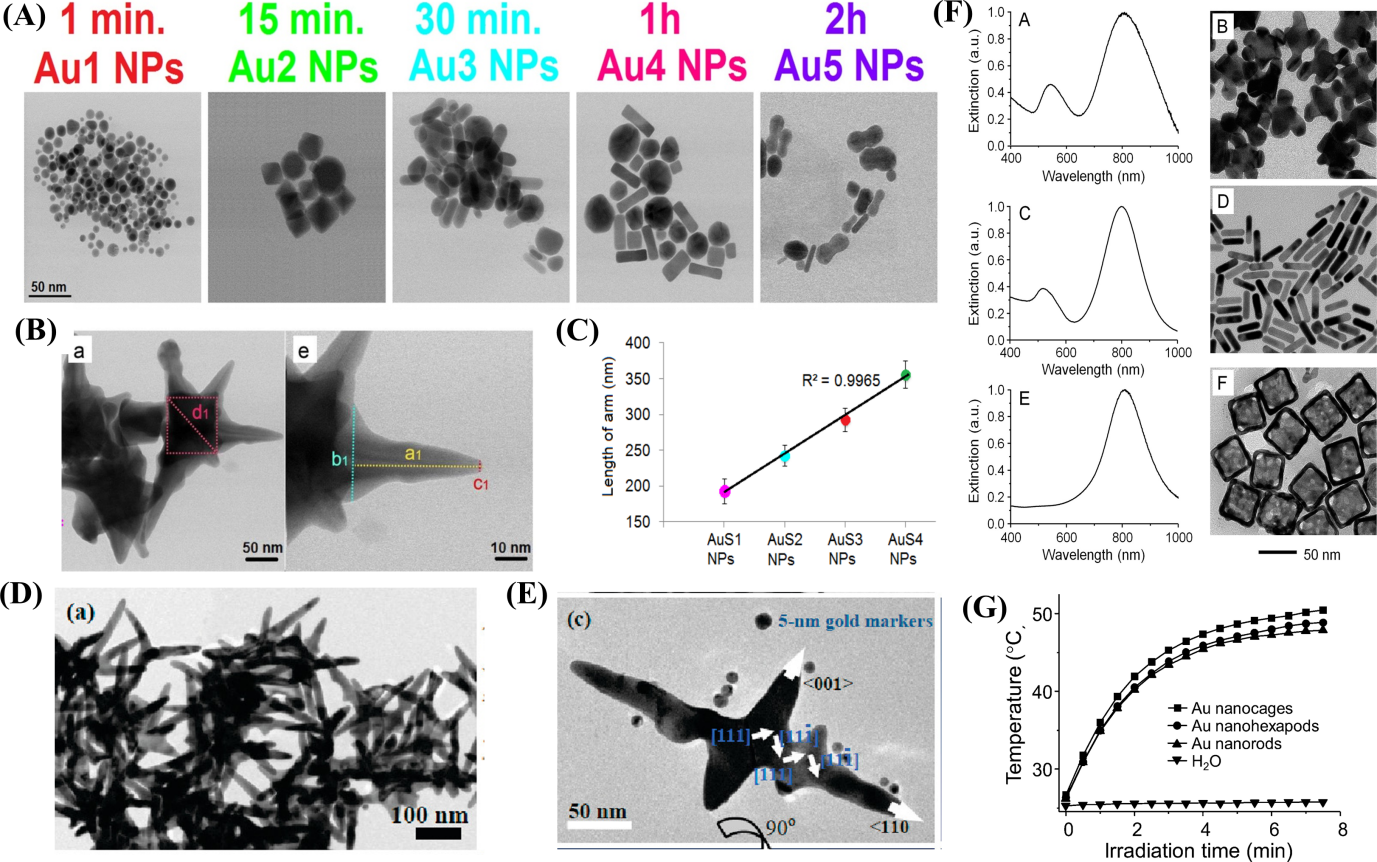
Shape-dependent photothermal properties. TEM image of gold nanostructures (A). (Figure 6A was adapted from [61], Photodiagnosis and Photodynamic Therapy, vol. 30, by J. Depciuch; M. Stec; M. Kandler; J. Baran; M. Parlinska-Wojtan, “From spherical to bone-shaped gold nanoparticles - Time factor in the formation of Au NPs, their optical and photothermal properties“, article no. 101670, Copyright (2020), with permission from Elsevier. This content is not subject to CC BY 4.0.) Scanning transmission electron image of Au nanostar (B). Limb length dependent photothermal activity of Au nanostar (C). (Figure 6B and C was adapted from [63] (© 2019 J. Depciuch et al., published by MDPI, distributed under the terms of the Creative Commons Attribution 4.0 International License, https://creativecommons.org/licenses/by/4.0).). TEM images of gold crosses (E). (Figure 6D and E was reprinted with permission from [62], Copyright 2011 American Chemical Society. This content is not subject to CC BY 4.0.). Absorbance and TEM images of different gold nanostructures (F). Irradiation time and temperature change plot for different gold nanostructures (G). (Figure 6F and G was reprinted with permission from [66], Copyright 2013 American Chemical Society. This content is not subject to CC BY 4.0.)

The photothermal properties of nanomaterials also depend on their morphology. It is possible to control the shape of nanoparticles through a number of available synthesis methods. Compared to the plasmonic properties of spherical gold nanoparticles and gold nanorods, gold nanostars have interesting properties because of their asymmetric spiky structure and narrow LSPR peaks [60]. Depciuch et al. synthesized three different gold nanostructures (spheres, rods, and bone-like rod structures) and compared their SPR peaks and their PCE properties. Though the SPR peaks were in the same wavelength range, the temperature increase was different (Figure 6A). It was also found that the bone-like structure produced the highest temperature of about 70 °C with 33% photo conversion efficiency [61].

Another fascinating class of gold nanostructures are branched or star-shaped architectonics [62]. Star-shaped nanoparticles contain a central core with sharp limbs. The electric field localized at the tip of the limbs will cause a dephasing of surface electrons. This energy will be transmitted to the atomic lattice and produce heat. In general, the production of heat highly depends on the ratio between absorbed and scattered light. This ratio is high in the case of the star architecture [63]. Because of their NIR absorption efficiency and large surface area, star-shaped nanoparticles have a higher PCE than regular nanostructures [64]. The LSPR peak and surface area of these nanomaterials can be increased by controlling the arm length and the number of arms [63] (Figure 6B,C). Lengthening the arms of nanostars could boost the material’s LSPR and photothermal activity even more [65]. Gold nanohexapods are another intriguing class of gold nanostructures. They consist of an octahedral core and six orthogonal arms. The absorbance region of this shape can be easily switched from green to the NIR region, resulting in outstanding photothermal properties [62]. Wang et al. investigated the shape-dependent photothermal activity of three different gold nanostructures (nanocages, nanorods, and nanohexapods) for photothermal therapy. All three nanostructures exhibited NIR absorption and could convert light into heat, with the gold nanohexapods having the highest efficiency [66] (Figure 6F,G). Branched gold nanostructures (Figure 6D,E) showed higher photothermal activity than spherical nanoparticles [62,64,67]. It was also shown that gold nanobipyramids have a more adjustable absorption wavelength and higher chemical stability than other gold nanostructures, which makes them suitable candidates for photothermal therapy [67].

Another well-known nanomaterial with photothermal properties are superparamagnetic iron oxide nanoparticles (SPIONs), which are commonly used as a magnetic hyperthermia agent. Because of the excellent absorption in the NIR region, they have been investigated also as photothermal agents [68]. Iron oxide nanoparticles have better stability and photothermal conversion efficiency than gold nanorods. However, only a limited number of studies have investigated the influence of size, shape, and coating material on the photothermal properties of SPIONs. Chu et al. investigated the PCE of three distinctly shaped (spheres, hexagons, and wires) iron oxide nanomaterials using a NIR laser. They found that the heat conversion of iron oxide nanoparticles is independent of their morphology and mainly depends on absorption wavelength and laser power [69]. Espinosa et al. reported the combined photothermal and magnetic hyperthermic effects of iron oxide nanoparticles on cancer cells generating two to five times more heat than from magnetic stimulation alone [70]. In another study, clustered iron oxide nanoparticles exhibited a higher PCE than separate iron oxide particles [71]. A study was conducted to analyse how a poly(acrylic acid) coating on iron oxide nanoparticles affected their photothermal activity. No significant difference in photothermal properties was observed between the coated and uncoated nanoparticles when exposed to 785 nm laser irradiation [72].

Cu-based nanoparticles are yet another class with many attractive optical features and good biocompatibility. Via the d–d energy band transition of Cu^2+^ ions, CuS nanoparticles can transform light into heat. Therefore, NIR-absorbing CuS NPs are being actively explored as novel photothermal agents. Again, the architectonics of the material plays a major role in the photo conversion efficiency. Flower-like structures of CuS nanoparticles showed high PCE due to multiple reflections of light enhancing photon absorption. Compared to hexagonal copper sulfide nanoparticles, the flower-like structure of CuS exhibited a 50% increase in photothermal conversion efficiency. By controlling the content of Cu in core–shell materials, such as Fe_3_O_4_@Cu_2−_*_x_*S, the photothermal properties can also be changed [44]. In addition, because of the high surface-to-volume ratio and highly functional surface ligands, the material can be easily conjugated with a wide variety of biomolecules on its surface, which is useful in applications such as photothermal therapy, imaging, and LFAs [73].

#### Composition-dependent photothermal properties

Recently, nanocomposite materials have been employed for the development of photothermal biosensors for the detection of cancer biomarkers or whole cancerous cells, antibiotic residues, and toxins [74]. The photothermal properties can also be enhanced by optimizing the elemental composition of the nanomaterials [44]. Generally, nanocomposite materials are developed by incorporating two or more compounds in a single particle, resulting in enhanced physicochemical properties of the final composite material [75]. For example, nanocomposite materials have been developed by combining noble metal nanoparticles (Au–Ag, Au–Pt, and Au–Ag–Pt) or hybrids of noble metals, metals, and/or carbon materials (Au–Fe, Au–carbon, and Au–Fe–carbon). The resultant nanocomposites exhibit properties of all constituents [74,76].

Au-based nanocomposites have been widely applied in the field of biosensor development because of their wide absorption and excellent light-to-heat conversion properties. Core–shell nanoparticles have a higher PCE than gold nanospheres [77]. Variations in the optical properties were observed by changing the composition of core–shell nanoparticles of silica and gold. A Au–Ag nanocomposite material developed by Lin et al. underwent structural changes, resulting in reduced photothermal conversion in the presence of H_2_S [78]. A multifunctional nanocomposite based on Au–Pt was developed by Li et al. for the detection and quantification of the cardiac disease-associated biomarker troponin I through photothermal and nanozymatic principles [79].

Fe_3_O_4_@Au core–shell nanostructures exhibit excellent photothermal conversion and magnetic properties, which have been studied regarding in vivo sensing and photothermal therapy. The high refractive index of Fe_3_O_4_ can be used to achieve strong NIR absorption. The addition of a magnetic part to nanosystems enables multifunctionality. Qiu et al. developed a plasmonic gold core with a MgFe_2_O_4_ shell to combine the magnetic and photothermal properties. After irradiation with a laser source for 4 min, there was a temperature increase up to 45.7 °C [80]. Fe_3_O_4_@Au nanocomposites showed a better PCE than their individual constituents and were applied for the detection of the antigen ochratoxin A (OTA) in a competitive LFA. The magnetic material in the nanocomposite was used for the preconcentration of antigen in the sample using a magnetic field and Au was used as a photothermal signal amplification probe. This method enabled the quantitative determination of OTA using a portable thermal camera with a limit of detection (LOD) of 0.12 pg·mL^−1^ [81].

Graphene has advantages such as high NIR absorption, large surface area, and biocompatibility. Also, it is known to enhance the signal intensity in SERS, albeit less efficiently than gold nanoparticles [82–83]. However, graphene can be used for photothermal applications. Using a hybrid formulation of gold and reduced graphene oxide (rGO), SERS imaging can be done along with photothermal therapy [84]. Recently, our group developed a multifunctional rGO–Au nanoscale architecture loaded with Raman dye and anticancer drugs for fluorescence/SERS imaging-guided breast cancer therapy. Under activation of a laser at 532 and 808 nm, the nanocomposite exhibited both photodynamic and photothermal properties. The same construct could be useful to design biosensors for the multimodal detection of analytes [85].

Recently, a catalytic and photothermal sensor was developed by Lu et al. for the detection of *Salmonella typhimurium* bacteria using Prussian blue and a Au composite. The results proved that photothermal signal amplification yielded a much higher sensitivity (10^1^ CFU·mL^−1^) than the colorimetric method (10^2^ CFU·mL^−1^) [86]. Before that, the same group developed a MoS_2_@Au nanocomposite for the photothermal detection of the same *Salmonella typhimurium* bacterium with a LOD of 10^2^ CFU·mL^−1^, which is lower than that of the Prussian blue–GNP composite [87]. From the above examples, it is clear that size, shape, and composition of a nanomaterial play significant roles in the photothermal properties and the efficiency of photothermal LFAs.

### Recent advances in photothermal nanomaterials in LFA

Many photothermal materials have been reported for therapeutic applications such as cancer therapy. However, their diagnostic applications are still limited, particularly regarding LFAs. Table 1 summarises some photothermal LFA applications. Gold-based photothermal materials, such as GNPs and gold nanorods, showed successful results in photothermal therapy and sensor applications. Generally, four main parameters need to be considered while developing a photothermal LFA system. The most important thing is the selection of the photothermal material; the material should exhibit strong light-to-heat conversion and good photostability. The structural and morphological properties of the selected material should ensure strong absorption in the NIR region to attain better penetration depth in the LFA nitrocellulose membrane and for efficient light-to-heat conversion. The surface chemistry of the selected photothermal material should facilitate the successful immobilization of biomolecules, such as antibodies, aptamers, peptides, or affinity molecules. Finally, an efficient thermal energy readout system is needed for the quantitative measurement of target analytes. Along with the above traits, the selected photothermal material should be well integrated in the assay membrane for a smooth flow of biomolecules.

**Table 1:** Summary of lateral flow assay applications based on the photothermal principle.

Sl.No	Technique	Tracer	Analyte	Sensitivity	LOD	Remarks	Ref

1	photothermal	r-GO	*E. coli* O157:H7	5 × 10^5^ to 5 × 10^7^ CFU·mL^−1^	5 × 10^5^ CFU·mL^−1^	photothermal signal amplification method demonstrated a tenfold sensitivity improvement over visual colour change	[27]
2	photothermal and SERS	DTNB dual layered Au@Pt@Ag NPs	DHEA	0.025 to 100.0 ng·mL^− 1^	0.42 ng·mL^− 1^	compared to GNP-based LFA, a 200-fold increase in sensitivity was obtained	[88]
3	photothermal	GNP	Cryp-tococcal antigen (CrAg)	*R*^2^ = 0.98	2.5 × 10^9^	analytical sensitivity improved 32-fold	[10]
4	photothermal	GNP	C-reactive protein	3 log_10_ to 6 log_10_	1.6 × 10^−3^ nM	compared to smaller GNPs, 100 nm GNP showed a 256-fold increase in sensitivity	[25]
5	photothermal	carboxylated graphene oxide	*Salmonella typhimurium*	10^3^ to 10^8^ CFU·mL^−1^	10^4^ CFU·mL^−1^	resistive temperature sensor used for effective capture of heat signal	[89]
6	magnetic and photothermal	Fe_3_O_4_-Au	ochratoxin A	1 pg·mL^−1^ to 1 μg·mL^−1^	0.12 pg·mL^−1^	Fe_3_O_4_@Au showed better PCE than Au and Fe, separately	[81]
7	photothermal and SERS	Au nanocage	alpha-fetoprotein and zearalenone	*R*^2^ > 0.99	0.25 ng·mL^−1^ and 2.3 ng·mL^−1^	5–6-fold better sensitivity than GNP-based lateral flow assay with 82–115% of recovery rate	[90]
8	photothermal and colorimetric	Fe@Fe_3_O_4_@NaYF4:Er3+/Yb3+/Nd3+ with black phosphorus	norfloxacin	5 to 2000 ng·mL^− 1^	45 pg·mL^−1^	achieved double the sensitivity levels by utilizing the built-in readout mechanism of a mobile phone	[91]

The photothermal conversion efficiency of gold nanoparticles in LFAs demonstrated by a cryptococcal antigen (CrAg) LFA kit showed a 32-fold increase in analytical sensitivity, which is comparable to the sensitivity level of standard laboratory techniques [10]. The analytical sensitivity can be further improved by tailoring nanomaterial architectonics, as discussed above. For instance, different nanoscale architectures of gold nanomaterials yield different photothermal efficiencies [55,79]. In LFA, gold nanoshells and gold nanorods showed, respectively, 4.6- and 36-fold higher photothermal conversion values than spherical gold nanoparticles [10].

**Figure 7:**
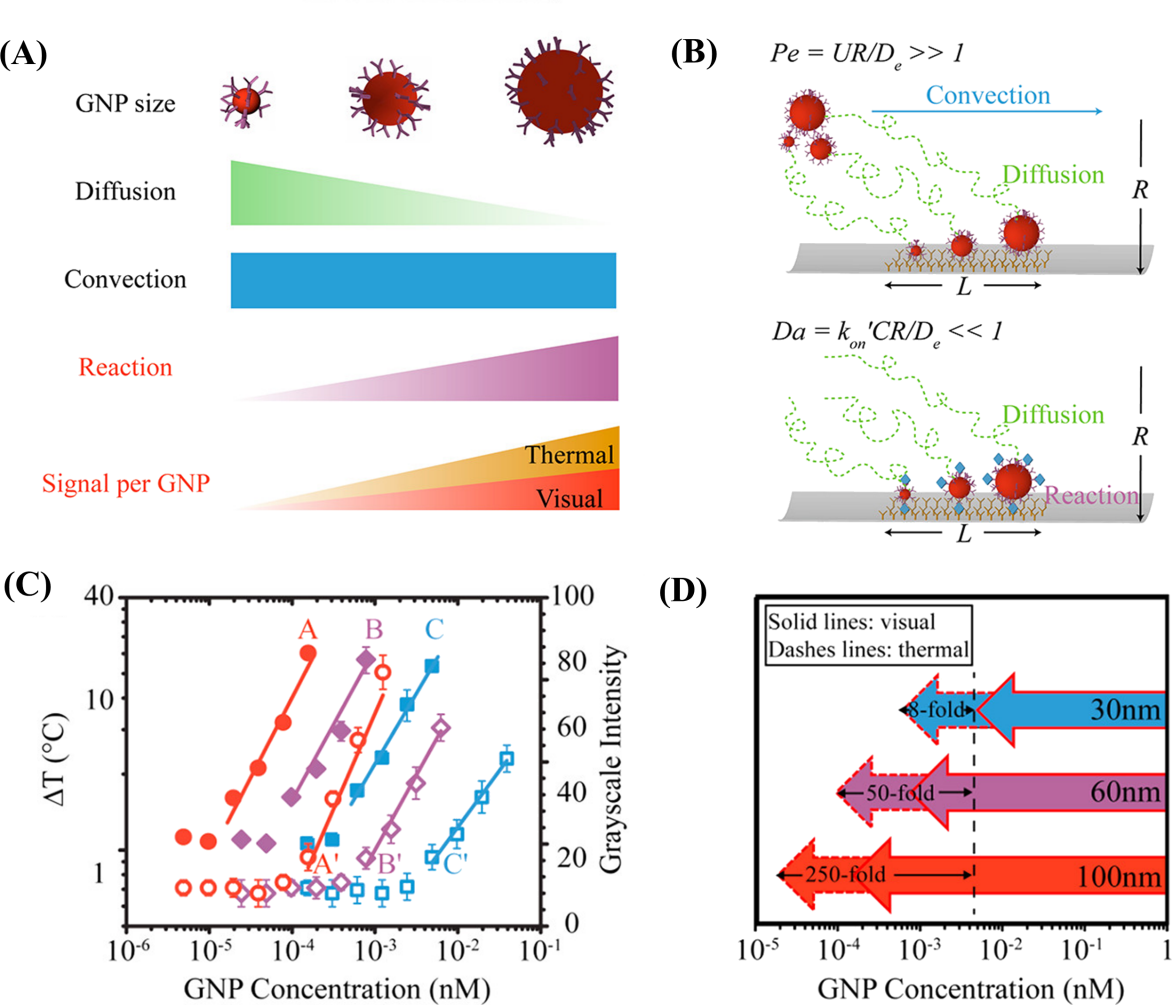
Influence of GNP size (30, 60, and 100 nm) on photothermal LFA (A). Comparison of Pe and Da on the LFA membrane. (Pe, the ratio between diffusion time and convection time), (Da, ratio between diffusion flux and reaction flux) (B). Comparison of visual and thermal signal (A, B and C stand for 100, 60, 30 nm sized GNP respectively: A, B and C correspond to thermal signal and A′, B′ and C′ correspond to visual signal) (C). Comparison of assay sensitivities between visual and thermal LFA using different-sized GNPs (D). (Figure 7A–D were adapted with permission from [25], Copyright 2017 American Chemical Society. This content is not subject to CC BY 4.0.)

Interesting research work was carried out by the Bischof group in 2017 to determine the impact of GNP size on LFA performance [25]. They compared three different sizes of GNPs (30, 60, and 100 nm) in order to determine both the colorimetric and photothermal effect on the LFA membrane (Figure 7A). The group considered C-reactive protein as a model antigen, and the result revealed that 100 nm GNPs had a better colorimetric and photothermal response than the smaller GNPs (Figure 7C). This increase in photothermal efficiency is attributed to the increased cross-sectional area of each particle. However, a further increase in the size of GNPs reduced the flow of nanoparticles through the nitrocellulose membrane pores and led to nonspecific binding of analytes and unwanted background signals (Figure 7B). In comparison to the visual method, the photothermal signal amplification technique allowed for the quantitative analysis of analytes with a sensitivity increased by a factor of almost 256 (Figure 7D). This observation underscores the importance of optimizing GNP size for maximizing sensitivity while mitigating potential drawbacks. This finding opens new possibilities for the highly sensitive and quantitative detection using LFAs, enhancing the applicability in various diagnostic tools. Based on the photothermal principle, very recently, Shirshahi et al. developed LFA strips for the detection of *E. coli* O157:H7 strains of bacteria. They used reduced graphene oxide as both tracer and photothermal signal amplification material. After the deposition of rGO on the test line, they irradiated it with 0.6 W·cm^−2^ power for 30 s, and the generated heat energy was captured using a IR thermal camera. The test result showed a tenfold increment in sensitivity compared to the visual colour change. Also it was possible to differentiate the targeted bacterial strain from six other strains of bacteria [27]. The integration of rGO into LFAs opens up exciting possibilities for developing ultrasensitive and selective diagnostic platforms, owing to the increased surface area and abundance of functional groups on the rGO surface.

In another study, carboxylated graphene oxide was used to develop photothermal strips for the diagnosis of *Salmonella typhimurium*. The authors used a conventional resistive temperature sensor to capture the heat signal from the strip and the detection limit was found to be 10^4^ CFU·mL^−1^ with 20 min of detection time [89]. Hu et al. previously demonstrated the effect of the gold nanoparticle architecture on the photothermal conversion efficiency in LFAs [90]. Gold nanocages (GNCs) exhibited higher photothermal conversion efficiency than other nanostructures, including gold nanospheres, shells, and stars. Using GNCs, they performed sandwich and competitive LFAs to detect, respectively, the macromolecule alpha-fetoprotein (AFP) and small-molecule zearalenone (ZEN). GNCs have a wide absorption spectrum (700–1000 nm); therefore, they can be excited at an IR wavelength of 808 nm rather than at the GNP excitation wavelength of 532 nm, which yields a lower photothermal effect. For AFP and ZEN, the LODs of the experiment were calculated as 0.25 and 2.3 ng·mL^−1^, respectively. The values were five to six times higher than the value of conventional LFA. They observed that the laser power also played a significant role in defining the sensitivity of the LFA. In addition, the photothermal effect of antibody conjugation on GNCs was studied. The antibody-conjugated GNCs did not show any observable change in temperature difference compared to bare GNCs. The photostability of GNCs on the LFA membrane was studied with five cycles of laser heating (2 min) and cooling under 808 nm irradiation. The GNCs showed the highest photostability between 88.6 and 90.9 °C and a loss of heat due to minimal photobleaching [90]. The combination of wide absorption spectrum, enhanced photothermal effect, and excellent photostability makes GNCs an exciting prospect for future diagnostic technologies. With further refinement and advancements, GNC-based photothermal assays could revolutionize the field of point-of-care diagnostics and reliable disease detection.

Recent studies have proven the applicability of multimodal LFAs for accurate and quantitative diagnosis. Yang et al. developed a multifunctional Au@Pt@Ag nanocomposite loaded with the Raman dye 5,5′-dithiobis-(2-nitrobenzoic acid) for the sensitive and quantitative detection of dehydroepiandrosterone (DHEA) through LFA. The developed nanocomposite combined colour signal, SERS, and photothermal properties, providing a platform for the multimodal detection of DHEA. The estimated LOD for the photothermal method was 0.42 ng·mL^−1^, which is higher than the value of the colour signal (1.0 ng·mL^−1^). The SERS mode showed higher sensitivity (0.013 ng·mL^−1^) than the photothermal LFA. However, the photothermal method showed a 200-fold higher sensitivity than a conventional gold nanoparticle-based LFA. Further, the developed strips were validated with real-field samples and the recovery rate of samples was found to be 96.7% to 106.7% for the photothermal method, which was equivalent to the recovery rate of the SERS method (98.2–105.2%) [88]. The integration of multiple modalities in a nanocomposite enhances the detection capabilities of LFAs, offering a promising approach for disease diagnosis and other analytical applications.

Shao et al. reported excellent photothermal properties of black phosphorous quantum dots and nanosheets [92]. The photothermal properties of two-dimensional black phosphorous nanosheets was first utilized by Ren et al. in LFAs for the sensitive detection of norfloxacin in tap water. The nanoscale complex of magnetic upconversion particles and black phosphorus has a characteristic brown colour that acts as a visual signal in the LFA and added photothermal properties to the assay. The LOD of this method was 45 pg·mL^−1^, which is two times higher than that of a GNP-based assay. Further, the authors developed an integrated system of mobile phone camera and laser for the quantitative measurement of analytes within 20 min [91]. The utilization of black phosphorous nanosheets in LFA showcases its potential for sensitive and visual detection of norfloxacin in tap water. Although the LOD is higher than that of the GNP-based assay, the development of an integrated system of mobile phone camera and laser provides a convenient and rapid solution for quantitative measurements using LFAs.

## Conclusion

We have discussed various nanoarchitectonics of photothermal nanomaterials for efficient photothermal conversion along with their applications in LFAs. The sensitivity of LFAs can be significantly enhanced by optimizing the nanoarchitectonics of nanomaterials to achieve superior light-to-heat conversion. We have briefly explained different photothermal nanomaterials, including noble metals, carbon-based materials, polymeric nanomaterials, and semiconductor materials, with their respective mechanism responsible for light-to-heat conversion. The photothermal conversion efficiency mainly depends on the absorption wavelength and the composition of the nanomaterials. The absorption wavelength should lie in the NIR region to obtain the maximum photothermal conversion. For plasmonic nanomaterials, this can be tuned by controlling the nanoscale architecture of these nanomaterials, such as size, shape, and structure. Furthermore, the light-to-heat conversion efficiency of carbon-based materials can be increased by functionalizing them with other nanostructures or biomolecules. Finally, we have reviewed research papers describing photothermal LFA and found that all of them noted an increase in sensitivity compared to traditional LFA. In addition to the photothermal properties, these nanomaterials have a high surface-to-volume ratio and numerous chemical functionalities on the surface, making them easier to functionalize with targeting agents such as antibodies and aptamers for LFAs. Therefore, photothermal LFAs are believed to play a significant role in the development of highly sensitive LFAs and other POCT devices.

## Data Availability

This manuscript is a review; the data presented here were all reproduced from published articles. The figures were either drawn by the authors or reproduced with permission.
